# Recombinant acetylxylan esterase of *Halalkalibacterium halodurans* NAH-Egypt: molecular and biochemical study

**DOI:** 10.1186/s13568-022-01476-w

**Published:** 2022-10-26

**Authors:** Amira M. Embaby, Hoda E. Mahmoud

**Affiliations:** Department of Biotechnology, Institute of Graduate Studies and Research, Alexandria University, 163 Horreya Avenue, P.O. Box 832, 21526 Chatby, Alexandria, Egypt

**Keywords:** *H. halodurans* NAH-Egypt, Recombinant AXE-HAS10, Structural modeling, Detergent stable, Metal ions stable, Alkalihalotolerant, Beechwood xylan hydrolysis

## Abstract

Acetylxylan esterase plays a crucial role in xylan hydrolysis as the acetyl side-groups restrict endoxylanase action by stearic hindrance. In this study, an acetylxylan esterase *(AXE-HAS10*: 960 bp & 319 a.a) putative ORF from *Halalkalibacterium halodurans* NAH-Egypt was extensively studied through heterologous overexpression in *Escherichia coli*, biochemical characterization, and structural modeling. The AXE-HAS10 tertiary structure was predicted by the Local Meta Threading Server. AXE-HAS10 belongs to the carbohydrate esterase Family 7. Purified to homogeneity AXE-HAS10 showed specific activity (36.99 U/mg), fold purification (11.42), and molecular mass (41.39 kDa). AXE-HAS10 showed optimal pH (8.5) and temperature (40 ^o^C). After 15 h of incubation at pH 7.0–9.0, AXE-HAS10 maintained 100% activity. After 120 min at 35 and 40 ^o^C, the retained activity was 80 and 50%, respectively. At 10 mM Mn^2+^, Fe^3+^, K^+^, and Ca^2+^ after 30 min, retained activity was 329 ± 15, 212 ± 5.2, 123 ± 1.4, and 120 ± 3.0%, respectively. After 30 min of preincubation with triton x-100, SDS, and CTAB at 0.1% (v/v), the retained activity was 150 ± 19, 88 ± 4, and 82 ± 7%, respectively. At 6.0 M NaCl after 30 min, retained activity was 58%. A 1.44-fold enhancement of beechwood xylan hydrolysis was achieved by AXE-HAS10 and *Penicillium chrysogenum* DSM105774 β-xylanase concurrently. Present data underpins AXE-HAS10 as a promising AXE for industrial exploitation.

**Supplementary Information:**

The online version contains supplementary material available at 10.1186/s13568-022-01476-w.

## Introduction

Acetylxylan esterases (AXEs: E.C. 3.1.1.72) are carbohydrate esterase enzymes that hydrolyze highly acetylated xylan polymers like hardwood xylans by breaking the ester bonds of the acetyl groups at positions 2 and/or 3 of the xylose moieties of natural acetylated xylan. During xylan hydrolysis by xylanase, the connected acetyl side residues in the xylan polymer interfere with enzyme accessibility to the xylan backbone via stearic hindrance. Because of the de-acetylation step mediated by AXEs, the xylan polymer would be more accessible to the activity of β-xylanases. The synergistic effect of the two enzymes β-xylanases and AXE would result in efficient xylan polymer hydrolysis and conversion to short chains of xylooligosaccharides and D-xylose (Malgas et al. 2019; Biely 2012; Hettiarachchi et al. 2019). The CAZy database (Carbohydrate-Active enZYmes Database), located on the server (http://www.cazy.org/), has classified the AXE as a member of carbohydrate esterases (CEs) that currently encompass 19 families, including acetyl xylan esterases (AXEs), chitin deacetylases, acetyl esterases, feruloyl esterases, peptidoglycan deacetylases, pectin methylesterases, pectin acetyl esterases, and glucuronoyl esterases (Biely 2012; Lombard et al., 2014). According to the update list of CEs in the CAZy database, AXE(s) are assigned to only 9 families: CE1-7, CE12, and CE16 in accordance with amino acid sequence similarity, protein structure, and substrate specificity. The first report addressing the discovery of microbial AXEs was that of Biely and co-workers, who discovered AXEs of fungal origin (Biely et al. 1985). Further reports have been deposited in publicly accessible databases highlighting the isolation of AXEs from a panel of microorganisms including fungi (e.g., *Trichoderma reesei* (Biely et al. 1997), *Volvariella volvacea* (Shaojun et al. 2007), *Aspergillus versicolor* (Prashant et al. 2016), *Penicillium chrysogenum* P33 (Yang et al. 2017), and *Aspergillus luchuensis* (Komiya et al. 2017)), and bacteria (e.g., *Streptomyces lividans* (Dupont et al. 1996), *Streptomyces albus* (Wenjing et al. 2012), *Bacillus subtilis* (Mitsushima et al. 1995; Vincent et al., 2003), *Bacillus pumilus* (Degrassi et al. 2000), *Thermotoga maritima* (Levisson et al. 2012), *Arthrobacter* sp. MTCC 5214 (Rakhee and Thasneem 2014), *Ochrovirga pacifica* (Hettiarachchi et al. 2019), and *Lactobacillus antri* (Min-Jeong et al., 2020)). Right now, the reported and well-characterized AXEs are considered thermostable AXEs. In contrast, a few reports have highlighted cold-adapted AXEs in the review literature so far.

Despite the promise of AXEs in the processing of hemicellulosic materials utilized in a wide range of industries, only a few AXEs produced from microbial sources have been studied and characterized. Through the NCBI Prokaryotic Genome Annotation Pipeline, the National Center for Biotechnology Information (NCBI) has begun a large number of whole microbial genome (WGS) sequencing studies, as well as the prediction/annotation of protein-coding sequences (PGAP). As a result, many microbial whole-genome sequences have been annotated with multiple putative AXE-encoding genes (predicted by ab initio gene prediction algorithms and homology-based methods). Consequently, both *in silico* and *non-in silico* sequence analyses are required to derive the structural-functional links for predicted AXEs.

So far, only two whole-genome sequencing records of *Halalkalibacterium halodurans* (previously known as *Bacillus halodurans*) strains, C-125 and LB-1, have been published in publicly accessible worldwide nucleotide databases. The WGS investigation of strain C-125 only revealed the existence of a putative AXE sequence. Kim et al. demonstrated that *Bacillus halodurans* C-125 AXE (BhAXE) was expressed heterologously in *E. coli* (Min-Jeong et al., 2018). However, Kim’s work did not go into detail on the AXE (BhAXE) of *B. halodurans* C-125, with only a few variables, including temperature and pH optima, being studied. Neither structural modelling nor in silico sequence analysis was investigated for BhAXE (Min-Jeong et al., 2018). *Halalkalibacterium halodurans* NAH-Egypt, an Egyptian, novel, and local strain previously isolated (Amer 2020), was identified by *16 S rRNA* gene sequence analysis (GB accession number: MG250277.1).

Biocatalysts are an appealing alternative for achieving chemical transformations in the framework of global needs for sustainability and clean industrial technologies (Bornscheuer et al. 2012; Shao et al., 2013). Enzymes are non-toxic, biodegradable, and effective biocatalysts with remarkable catalytic features. They provide high levels of safety, reduced energy usage, and an overall ecologically benign manufacturing process (Wang et al. 2012). Extremophiles, particularly microorganisms living in cold conditions, have become a very intriguing source for the identification and isolation of novel cold-active enzymes (Feller 2013). Cold-active enzymes enhance catalytic efficiency by providing structural flexibility at or near the active regions at low temperatures. Inevitably, this accomplishment appears to be coupled with a reduction in thermal stability (Mangiagalli and Lotti 2021). Moreover, enhanced structural flexibility may lead to broad substrate specificity for a variety of diverse substrates, a concept called “substrate promiscuity“ (Santiago et al. 2016). Cold-active enzymes have become promising targets for commercial applications in detergency, waste bioremediation, molecular biology, and the medical, pharmaceutical, and food industries thanks to their properties (Sondavid et al. 2020; Mangiagalli et al. 2020). Because they could minimize the energy cost of a reaction by significantly reducing the proper temperature without having to sacrifice enzyme activity, attenuate side-unwanted reactions that take place at elevated temperatures, and merely be inhibited because of thermoliability, which is of major interest in the food industry for ignoring the use of chemical-based inactivation (Sarmiento et al. 2015).

At most, at the strain level, the variety among members of the same species would impose different fingerprints on the features of the same kind of enzyme produced by this diverse panel of bacterial strains. However, the production of AXEs from their wild-type producers is restricted to a low level of gene expression that would impose a low level of enzyme activity. Furthermore, as a result of the industrial potential and scarcity of AXEs in the commercialized enzymes sector, there is an urgent and increased demand for novel AXEs with robust properties in the global enzyme markets. From this perspective, the current work’s goal is to clone, heterologously express in *E. coli*, and fully characterize the AXE from the Egyptian novel strain *Halalkalibacterium halodurans* NAH-Egypt. In addition, the predicted secondary, tertiary, and quaternary structures of the enzyme were built. To the best of the authors’ knowledge, this is the first report to underline recombinant cold-adapted, detergent-stable, metal ion-stable, and alkali-halotolerant AXE from *Halalkalibacterium halodurans.*

## Materials and methods

### Bacterial strains, cultivating conditions, vectors, and reagents

In this study, genomic DNA from *Halalkalibacterium halodurans* NAH-Egypt, formerly *Bacillus halodurans* NAH-Egypt, was utilized to isolate the gene encoding an acetylxylan esterase (AXE-HAS10). *Halalkalibacterium halodurans* NAH-Egypt was previously isolated and identified by *16 S rRNA* sequence analysis (Amer 2020). The *16 S rRNA* gene nucleotide sequence of *Halalkalibacterium halodurans* NAH-Egypt was deposited in GenBank under the accession number MG250277.1. The *Escherichia coli* DH5α strain and the *E. coli* BL21 (DE3) Rosetta strain were the cloning host and the expression host, respectively, used in this study. The pGEM^®^-T Easy vector (Promega Co., USA) and the pET-28a (+) vector (GenScript Co., USA) were used as the cloning and the expression vectors, respectively. A polymerase chain reaction (PCR) was performed using My Taq^™^ Mix (Bioline, USA). PCR products were purified using the GeneJET PCR Purification Kit (Thermo Fischer Scientific Co., USA). The GeneJET Plasmid Miniprep Kit (Thermo Fischer Scientific Co., USA) was used for plasmid extraction. Substrates used for the enzyme assay were *p*-nitrophenyl acetate (*p*-NP-C2), *p*-nitrophenyl butyrate (*p*-NP-C4), *p*-nitrophenyl caproate (*p*-NP-C6), *p*-nitrophenyl caprylate (*p*-NP-C8), and *p*-nitrophenyl laurate (*p*-NP-C12), and were purchased from Sigma-Aldrich Co., St. Louis, USA. D-xylose, 3,5-Dinitrosalicylic acid, and imidazole were purchased from Loba Chemie PVT, Mumbai, India. Isopropyl-β-D-1-thiogalactopyranoside (IPTG), 5-bromo-4-chloro-3-indolyl-β-D-galactopyranoside (x-gal), agarose, 1 Kbp DNA ladder, protein ladder, and kanamycin were purchased from Bioline, USA. The synergistic effect of AXE-HAS10 on xylan degradation was confirmed by partially purified β-xylanase of *Penicillium chrysogenum* Strain A3 DSM105774 (Matrawy et al. 2021) using beechwood xylan (Chemie PVT, Mumbai, India) as the substrate.

### Isolation and PCR cloning of AXE-HAS10 into pGEM®-T Easy vector

The putative acetylxylan esterase gene (locus _tag = AYT26_RS16720 and protein_id WP_010899467.1) of *Halalkalibacterium halodurans* C-125 (accession number of complete genome: NC_002570.2) was used to design the primer sets: AXE-HAS10-Fw 5’- *GGATCC*ATGATGCCACTAATAGACATGCCGT-3’ and AXE-HAS10-Rv 5’-*CTCGAG*TTAGAGATCAGATAAAAATTGAAAAATCC-3’. The italic sequences refer to recognition sites for the restriction enzymes: *Bam*H1 and *Xho*1, respectively. These primer sets were used to PCR amplify the full open reading frame (ORF) of acetylxylan esterase designated AXE-AHS10 from *Halalkalibacterium halodurans* strain NAH-Egypt. The composition of the PCR mixture and the PCR conditions were settled in Table S1. The amplified PCR product of 960 bp was purified using the GeneJET PCR Purification Kit. Then the purified PCR product was ligated into the linearized pGEM®-T Easy vector at 4 ^o^C for overnight according to the instructions of the manufacturer. The pGEM®-T/*AXE-HAS10* construct was transformed into chemically competent *E. coli* DH5α cells according to a previously reported procedure (Sambrook et al. 1989). Some randomly selected colonies were used to extract the recombinant vector pGEM®-T/*AXE-HAS10* using the GeneJET Plasmid Miniprep Kit according to the instructions of the manufacturer. The presence of the nucleotide sequence of the insert AXE-HAS10 in the recombinant plasmid pGEM®-T/*AXE-HAS10* was verified by DNA sequencing (Macrogen, Korea) using the universal plasmid primer sets Sp6 /T7.

### Construction of recombinant pET-28a (+)/AXE-HAS10

The recombinant plasmid pET-28a (+)/AXE-HAS10 was constructed by GenScript Co., USA (Order: U3326EL100 _2) using BamH1 and Xho1 restriction enzymes. The construct pET-28a (+)/AXE-HAS10 was transformed into chemically competent E. coli BL21 (DE3) Rosetta cells according to a previously reported protocol (Sambrook et al. 1989).

### Overexpression and purification of recombinant AXE-HAS10

The transformants *E. coli* BL21 (DE3) Rosetta cells harbouring the plasmid construct pET-28a (+)/*AXE-HAS10* were grown in LB broth supplemented with kanamycin at a final concentration of 34 µg/mL at 37℃ with an agitation speed of 180 rpm until the OD600 reached 0.6-08, and then IPTG was added to a final concentration of 1 mM. After incubation at 37℃ for 16 h, harvesting of the induced cells was performed by centrifugation at 5, 000 ×g for 5 min. Cell breakage by sonication (Fisher Brand TM Sound Enclosure, Thermo Fisher Scientific Co., USA) was performed according to a previously reported procedure (Mahmoud et al. 2021; Abady et al. 2022). The soluble supernatant of the cell lysate was maintained at -20 °C until further processing.

The purification of the recombinant AXE-HAS10 was performed according to a previously reported procedure (Mahmoud et al. 2021) using a Ni^2+^-NTA affinity matrix. The eluted fractions showing protein content (evidenced by absorbance at 280 nm) were subjected to dialysis using a 14, 000 Da membrane cut-off dialysis bag against 50 mM phosphate buffer, pH 7.5 at 4 ^o^C. The activity of the dialyzed recombinant AXE-HAS10 was performed using *p*-NP-C2 as the substrate.

### Protein determination

The protein content in the crude soluble cell lysate and the purified fraction was determined using the Bradford method using Coomassie Brilliant Blue G-250 (Bradford 1976). A standard curve of bovine serum albumin was established.

### SDS-PAGE

Sodium dodecyl sulfate polyacrylamide gel electrophoresis (10%) was performed to examine all protein fractions during the purification steps using the Laemmli method (Laemmli 1970).

### AXE-HAS10 enzyme assay, substrate specificity, and kinetics

The activity of AXE-HAS10 was estimated using five *p*-nitrophenyl esters (*p*-NP-C2,, *p*-NP-C4, *p*-NP-C6, *p*-NP-C8, and *p*-NP-C12 as the substrates (Bahiru et al. 2020), AXE-HAS10 can hydrolyze the ester bond in -*p*-nitrophenyl esters that would result in releasing the yellow-colored *p*-nitrophenol (*p* -NP). All enzyme assays performed with *p*-nitrophenyl esters were estimated by measuring the absorption of the yellow-colored *p*-NP at 410 nm. A standard curve of *p*-NP was established to determine the extinction coefficient of *p*-NP. One unit is defined as the amount of an enzyme that releases 1 µmol of *p*-nitrophenol (*p*-NP) per minute under the stated assay conditions (Cheeseman et al. 2001). Unless otherwise stated, the reaction mixture (1 mL) contained *p*-nitrophenyl ester substrate at a final concentration of 0.3 mM, 30 mM Tris-HCl, pH 8.0, and recombinant AXE-HAS10 in either crude or purified status. All assays were performed in triplicate. The *K*_m_ and *V*_max_ for purified AXE-HAS10 were estimated using *p*-NP-C2. The Lineweaver-Burk plot was drawn using the Hyper32 program.

### Biochemical characterization of purified to homogeneity AXE-HAS10

All enzyme biochemical characterization reactions were performed using *p*-NP-C2 as the substrate. All reactions were conducted in triplicates.

### Optimum pH and temperature

The optimum pH was determined using the following overlapping buffer system: 50 mM acetate buffer for pH (s) 3.0, 4.0, 5.6, 50 mM citrate buffer for pH(s) 5.6, 6.0 & 50 mM phosphate buffer for pH(s) 6.0, 7.0, 8.0 & 50 mM Tris-HCl buffer for pH(s) 7.0, 8.0, 8.5, 9.0 & 50 mM Glycine-NaOH buffer for pH(s) 9.0, 10.0, 11.0, and 50 mM carbonate buffer for pH(s) 10, 10.6. The optimal temperature was determined at different temperatures ranging from 5 to 60 °C.

### Effect of temperature and pH on AXE-HAS10 stability

For temperature stability experiments, the purified enzyme was pre-incubated in 50 mM Tris-HCl, pH 8.0 at different temperatures of 5, 10, 15, 20, 25, 30, 35, and 40 ^o^C over various time intervals of 30, 60, and 120 min each. Then, the enzyme activity was assayed at the optimal pH and optimal temperature. Then, residual activity was determined at each pre-incubation temperature.

For the pH stability experiments, the purified enzyme was pre-incubated in the aforementioned buffers for 15 h at 4 ^o^C. After termination of the incubation time, enzyme assays were performed at optimal pH and temperature. Control reactions were conducted at each pH. Residual activity was determined at each pH.

### Effect of metal ions, β-mercaptoethanol, EDTA, detergents, organic solvents, and NaCl on AXE-HAS10 stability

AXE-HAS10 was pre-incubated for 30 min in the presence of Ca^2+^, Mg^2+^, Fe^3+^, Mn^2+^, Cu^2+^, Zn^2+^, Mo^2+^, K^+^, β-mercaptoethanol and ethylene diamine tetra -acetic acid (EDTA) at two concentrations, 5 and 10 mM each. For the effect of polar and non-polar solvents on AXE-HAS10 stability, the enzyme was pre-incubated in the presence of the following solvents (hexane, butanol, acetone, glycerol, isopropanol, ethanol, methanol, and dimethyl sulfoxide (DMSO)) at two concentrations of 10 and 20% (v/v) each. For the effect of cationic, anionic, and non-ionic detergents on AXE-HAS10 stability, the enzyme was pre-incubated for 30 min in the presence of Cetyl-trimethylammonium bromide (CTAB), sodium dodecyl sulfate (SDS), and Triton X-100 at two concentrations of 0.01 and 0.02% (v/v) each. Moreover, the enzyme was pre-incubated for 30 min in the presence of different concentrations (0.5-6.0 M) of NaCl.

### Effect of PMSF

The effect of paramethyl sulfonyl fluoride (PMSF) on AXE-HAS10 was tested in two concentrations, 1.0 and 3.0 mM. The enzyme was pre-incubated for 30 min with PMSF at each tested concentration. Then, the residual activity was estimated.

An enzyme test without pretreatment was used as a control reaction. The residual activity was determined in the case of each tested effector.

### Synergistic effect of AXE-HAS10

The synergistic effect of AXE-HAS10 on the hydrolysis of beechwood xylan by β-xylanase of *Penicillium chrysogenum* Strain A3 DSM105774 (Matrawy et al. 2021) was evaluated by measuring the reducing sugar xylose released using a 3,5-Dinitrosalicylic acid (DNS) method (Miller 1959). A standard curve of D-xylose was established. The β-xylanase of *P. chrysogenum* Strain A3 DSM105774 was partially purified using fractional ammonium sulfate precipitation of 60–80% followed by dialysis. The reaction mixture was settled in an Eppendorf tube containing 1% (w/v) beechwood xylan (Bioloba LLC, Mumbai City, India) in 50 mM phosphate buffer (pH 7.0), 10 U β-xylanase, and 3.0 U AXE-HAS10. The reaction mixture was incubated at 40 °C, and the activity was checked at 30 min intervals for 2.5 h by measuring the amount of reducing sugars.

### In silico AXE-HAS10 sequence analysis

The Signal IP 5.0 server (http://www.cbs.dtu.dk/services/SignalP/) (Petersen et al. 2011) was used to predict the N-terminal signal peptide of the AXE-HAS10 amino acid sequence. The translated protein amino acid sequence of AXE-HAS10 was obtained via Expasy, the Swiss Bioinformatics Resource Portal, located on the server (https://web.expasy.org/translate/). The nucleotide sequence of AXE-HAS10 and its translated protein amino acid sequence were searched against the non-redundant nucleotide collection database and UniProtKB/Swiss-Prot (Swissprot) using the BLASTN and BLASTP online programs, respectively. The secondary structure of the translated AXE-HAS10 protein was predicted using the SAS server (https://www.ebi.ac.uk/thornton-srv/databases/sas/). The multiple sequence alignment of the AXE-HAS10 amino acid sequence and those of acetylxylan esterases (AXEs) from other species and the phylogenetic tree depicting the evolutionary relationships of aligned sequences were done by CLC Sequence Viewer 8.0. The three-dimensional (3D) structure of the AXE-HAS10 protein was predicted using the online Local Meta-Threading Server (LOMETS3) located at the server (https://zhanggroup.org/LOMETS/) (Wei et al. 2019). The PyMOL (Schrödinger, LLC, Portland, OR) was used to display the molecular image of the predicted 3D structure of AXE-HAS10 included in the PDB file, which was retrieved from the output of the LOMTS3 server. The theoretical pI, molecular mass, and amino acid composition of AXE-HAS10 were inferred from the webtool ProtParam located at the server https://web.expasy.org/protparam/. The presence of any transmembrane helices in the AXE-HAS10 protein was predicted by the three programs: TMHMM2.0 (https://services.healthtech.dtu.dk/service.php?TMHMM-2.0/), SOSUI (“https://harrier.nagahama-i-bio.ac.jp/sosui/mobile/ ”) (Mitaku 2002), and PHOBIUS (“https://www.ebi.ac.uk/Tools/pfa/phobius”).

## Results

### AEX-HAS10 gene isolation and sequence analysis

**Fig. 1:**
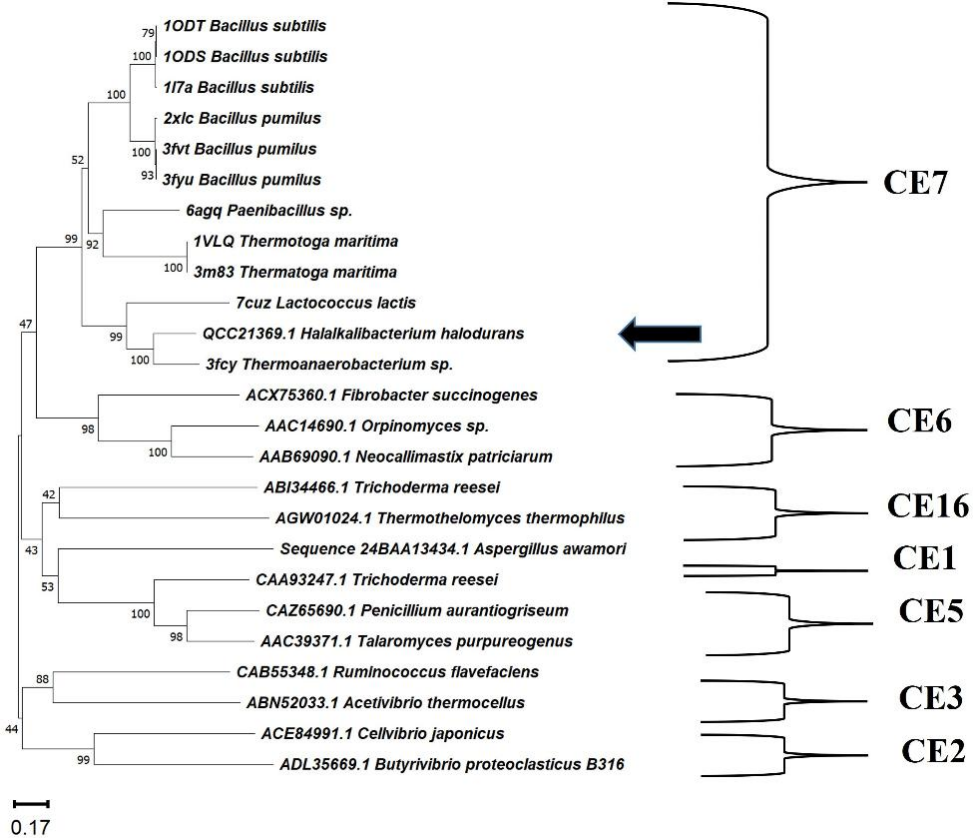
Neighbor-joining phylogenetic tree, constructed by MEGA 11.0, showing the genetic relatedness of AXE-HAS10 (accession number: QCC21369.1) deduced amino acid sequence of *Halalkalibacterium halodurans* strain NAH-Egypt (indicated by an arrow) with respect to other AXE(s) from other species. Numbers on branch nodes represent bootstrap values (1000 re-samplings)

**Fig. 2:**
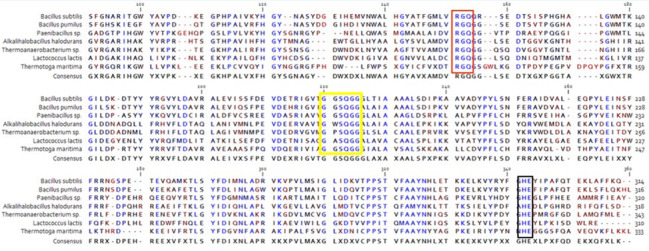
Multiple alignment of AXE-HAS10 with other related CE7 family members displaying the GxSxG (green rectangle), -RGQ- (yellow stars), and -HE- (green stars) conserved motifs performed with CLC Sequence Viewer 8.0. The presented acetylxylan esterases are *B. subtilis* (accession number: 1L7A), *B. pumilus* (accession number: 2xlb), *Paenibacillus* sp. (accession number: 6AGQ_A), *Halalkalibacterium halodurans* (accession number: QCC21369.1 (AXE-HAS10)), *Thermoanaerobacterium* sp. (accession number: 3FCY), *Lactococcus lactis* (accession number: 7CUZ_A), and *Thermotoga maritima* (accession number: 1VLQ).

The full length of the *acetylxylan esterase* ORF (960 bp) was successfully amplified by PCR from the locally isolated *Halalkalibacterium halodurans* strain NAH-Egypt using the primer set (AXE-HAS10-Fw/AXE-HAS10-Rv), designed based on the putative acetylxylan esterase (locus_tag = AYT26_RS16720 and protein_id WP_010899467.1) of *Halalkalibacterium halodurans* C125 (accession number of the complete genome: NC_002570.2). A BLASTn similarity search of the obtained nucleotide sequence against the refseq representative genomes (RefSeq representative genomes) database revealed that the query sequence had a significant similarity with the locus _tag="AYT26_RS16720 in *Halalkalibacterium halodurans* C125 (accession number of complete genome: NC_002570.2). Hence, the obtained nucleotide sequence of the laboratory isolated gene was deposited in GenBank under the accession number MK531136.1 and was named *AXE-HAS10*. A BLASTp similarity search against the RefSeq Select proteins (refseq select) database and the Protein Data Bank (PDB) database revealed that the obtained deduced amino acid sequence of AXE-AHS10 shared significant similarity with other acetylxylan esterases from both closely and distally related species as follows: WP_010899467.1 (*Halalkalibacterium halodurans* C125, 100% identity), WP_134229671.1 (*Halalkalibacterium halodurans* DSM497, 99.37% identity), 3FCY_A (*Thermoanerobacterium saccharolyticum*, 63.32% identity), 7CUZ_A (*Lactococcus lactis*, 41.58% identity), 3FVR_A (*Bacillus pumilus*, 37.85% identity), and 6AGQ_A (*Paenibacillus* sp. R4, 36.88% identity). Results of AXE-HAS10 amino acid sequence analysis through the InterPro online programme of EMBL-EBI revealed its affiliation to family CE7 (IPR039069). Amino acid sequence analysis of AXE-HAS10 through the Pfam online programme of EMBL-EBI deduced its affiliation to family AXE1 and the CL0028 clan. Twenty amino acid sequences of acetylxylan esterases belonging to family CE7 from other species were retrieved from InterPro to construct the phylogenetic tree depicted in Fig. 1. The proteins in family CE7 were distinguished by a set of consensus sequence motifs (G-x-S-x-G, –RGQ–, and –HE–) common in carbohydrate esterases (Fig. 2). The acetylxylan esterases’ typical motifs G-x-S-x-G, –RGQ–, and –HE– were found in AXE-HAS10 at amino acid positions 181–185, 118–120, and 302–304, respectively. The catalytic triad of AXE-HAS10, shown in Fig. 2, was located at Ser^183^ (embedded in the pentapeptide motif), Asp^273^, and His ^302^. The translated amino acid sequence of AXE-HAS10 consisted of 319 amino acid residues without any detectable signal peptide as inferred from the Signal IP-5.0 Server. AXE-HAS10 was not a transmembrane protein, inferred from the analysis of its protein sequence through three online servers: TMHMM2.0, SOSUI, and PHOBIUS. The predicted molecular mass and theoretical pI of AXE-HAS10 were 36.5 kDa and 5.67, respectively.

### Purification and expression of recombinant AXE-HAS10

**Fig. 3:**
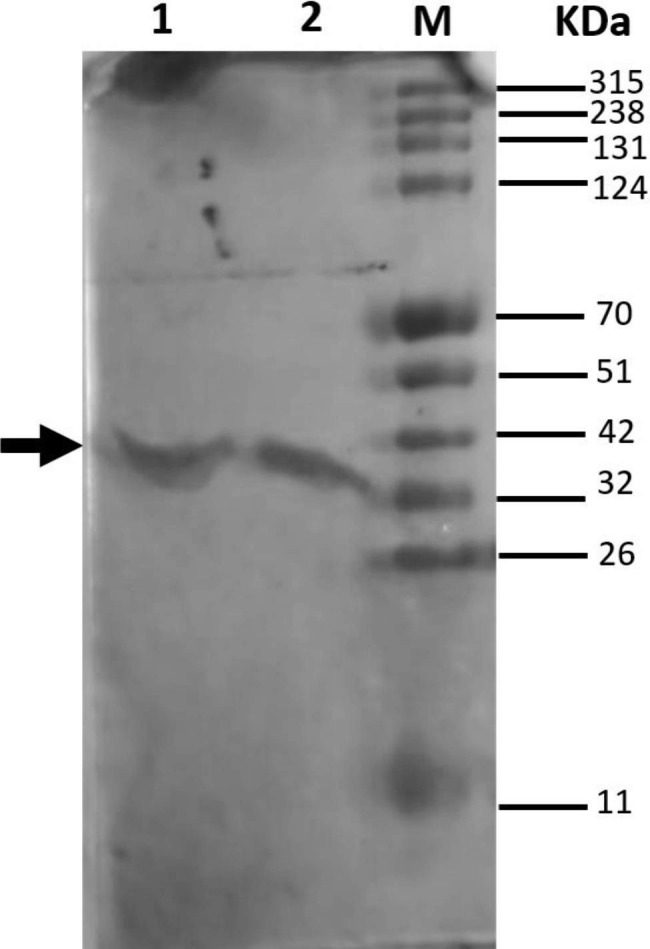
SDS-PAGE (10%) showing the recombinant expressed AXE-HAS10 during the stages of purification using Ni^2+^-affinity agarose matrix.: Lane M: protein ladder in kDa. Lanes 1 & 2 purified AXE-HAS10 after elution from the Ni^2+^ -affinity agarose matrix column using the elution buffer (50 mM Tris- HCl, pH 8.0, 500 mM imidazole) indicated by a sold arrow

The recombinant AXE-HAS10 was successfully expressed in frame as a fusion protein tagged with 6-his residues (Fig. 3). The recombinant AXE-HAS10 was purified to homogeneity using Ni^2+^affinity chromatography. The purified recombinant enzyme had specific activity, fold purification, and recovery of 36.99 U/mg, 11.45, and 71.84%, respectively (Table 1). As concluded from 10% SDS-PAGE (Fig. 3), the purified recombinant AXE-HAS10 displayed a molecular mass of approximately 41.39 kDa.

**Table 1:** Purification table and kinetic parameters for hydrolysis of *p*-NP-C2 by recombinant AXE-HAS10

Status	Vol. (mL)	Total units	Total mg protein	Specific activity (U/mg)	Fold	Yield (%)	*K*_*m*_ (mM)	*k*_*cat*_ (s^− 1^)	*k*_*cat*_*/K*_*m*_ (s^− 1^._mM_^−1^)
Crude soluble cell lysate	1.0	43.08	13.30	3.23	1.00	100.00	ND	ND	ND
Ni^2+^ affinity chromatography	9.0	30.95	0.837	36.99	11.450	71.84	0.096	63.06	6.571 × 10^2^

ND: not determined

### Biochemical characterization of recombinant AXE-HAS10

**Fig. 4:**
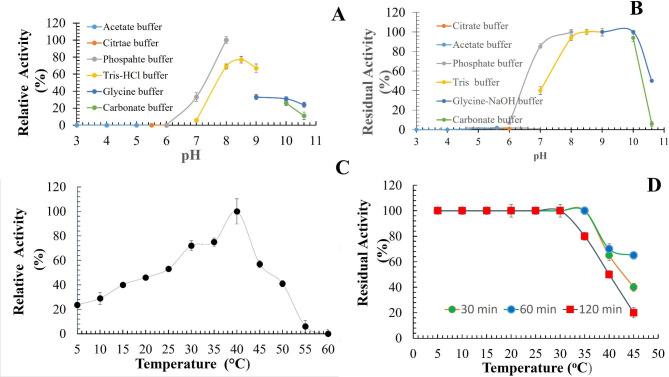
pH and temperature profiles of purified to homogeneity AXE-HAS10. A: AXE-HAS10 activity at different pH (s). B: AXE-HAS10 activity after 15 h preincubation at different pH (s). C: AXE-HAS10 activity at different temperatures. D: AXE-HAS10 activity after 30, 60, and 120 min preincubation at different temperatures. Values are the mean of three readings ± standard error

Recombinant AXE-HAS10’s optimal activity was at pH 8.5 and 40 °C (Fig. 4 A and C). The enzyme maintained 85% of its activity after pre-incubation of the enzyme at pH 7.0 for 15 h. However, 100 and 100% of the enzyme activity could be retained after pre-incubation of the enzyme at pH 8.0 and 9.0 for 15 h (Fig. 4B ). The enzyme could retain approximately 100% of its activity after pre-incubation at temperatures ranging from 5 to 30 ^o^C for 30, 60, and 120 min for each tested temperature without any detectable significant difference (Fig. 4D). A significant decline in the enzyme activity after 120 min at 35 and 40 ^o^C was noticed; 80 and 50%, respectively (Fig. 4D). The enzyme activity dramatically declined (approximately 18%) after pre-incubation at 45 ^o^C for 120 min (Fig. 4D).

**Fig. 5:**
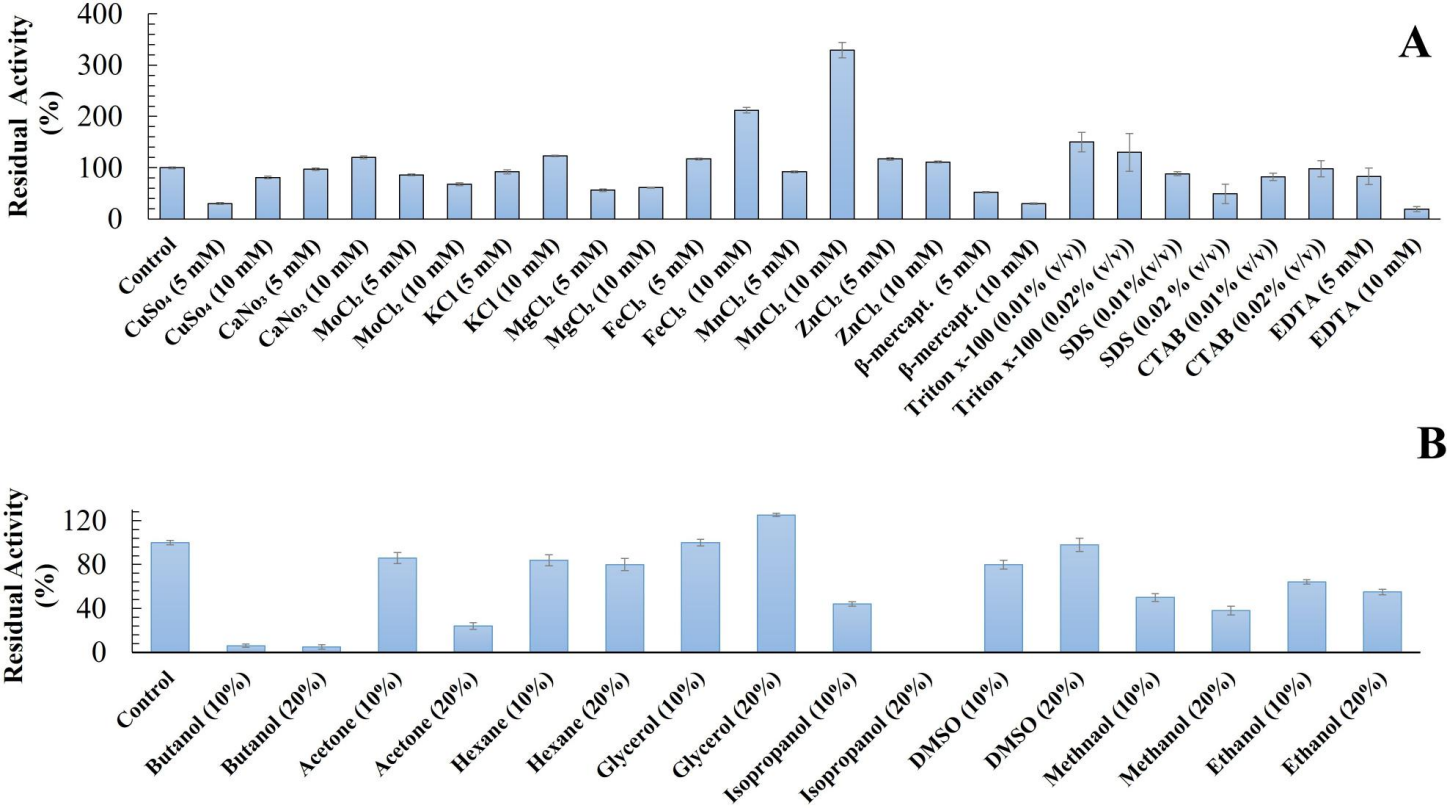
A: Effect of metal ions, β-mercaptoethnaol, EDTA, SDS, CTAB, and Triton x-100 on the stability of purified to homogeneity AXE-HAS10 after pre-incubation of the purified AXE-HAS10 with each effector for 30 min. B: Effect of some polar and non-polar solvents on the stability of purified to homogeneity AXE-HAS10 after pre-incubation of the purified AXE-HAS10 with each effector for 30 min. Values are the mean of three readings ± standard error. Control (set to be 100%) is the enzyme activity without treatment with an effector

The influence of various metal ions on the activity of the purified AXE-HAS10 was investigated (Fig. 5 A). The activity of the purified AXE-HAS10 was impressively enhanced by Mn^2+^, Fe^3+^, K^+^, Ca^2+^, and Zn^2+^ at 10 mM, retaining residual activity of 329 ± 15, 212 ± 5.2, 123 ± 1.4, 120 ± 3.0, and 111 ± 1.6%, respectively. Conversely, the activity of the purified enzyme was inhibited at both tested concentrations by Cu^2+^, Mo^2+^, and Mg^2+^. β-mercaptoethanol significantly inhibited the enzyme at both tested concentrations (Fig. 5 A).

The resistance of AXE-HAS10 against various detergents was studied (Fig. 5 A). Amazingly, the activity of the purified AXE-HAS10 was significantly enhanced by Triton x-100 at 0.01% (v/v) to 150 ± 19%. In contrast, neither an inhibitory nor a stimulatory effect on enzyme activity was noted in the presence of CTAB at both tested concentrations. On the other hand, the purified AXE-HAS10 exhibited partial resistance towards SDS, maintaining more than 50% of its initial activity at both tested concentrations. For the effect of EDTA, it showed an inhibitory effect in its two concentrations, 5 and 10 mM, on AXE-HAS10, retaining 83 ± 16 and 19.23 ± 5% residual activity, respectively (Fig. 5 A).

Regarding the polar and non-polar solvents, purified AXE-HAS10 was sensitive to all studied solvents except for glycerol (Fig. 5B). It was potently inhibited by butanol, acetone, isopropanol, and methanol at a concentration of 20% (v/v). However, it was slightly inhibited by hexane and DMSO at both tested concentrations, retaining approximately 81 ± 0.5% and 82 ± 2.2% of initial activity, respectively. Meanwhile, a 50% reduction in the enzyme activity was realized in the presence of isopropanol, methanol, and ethanol at concentrations of 10% (v/v), maintaining 47 ± 2.6, 52%± 1.0, and 65 ± 0.9%, respectively.

**Fig. 6:**
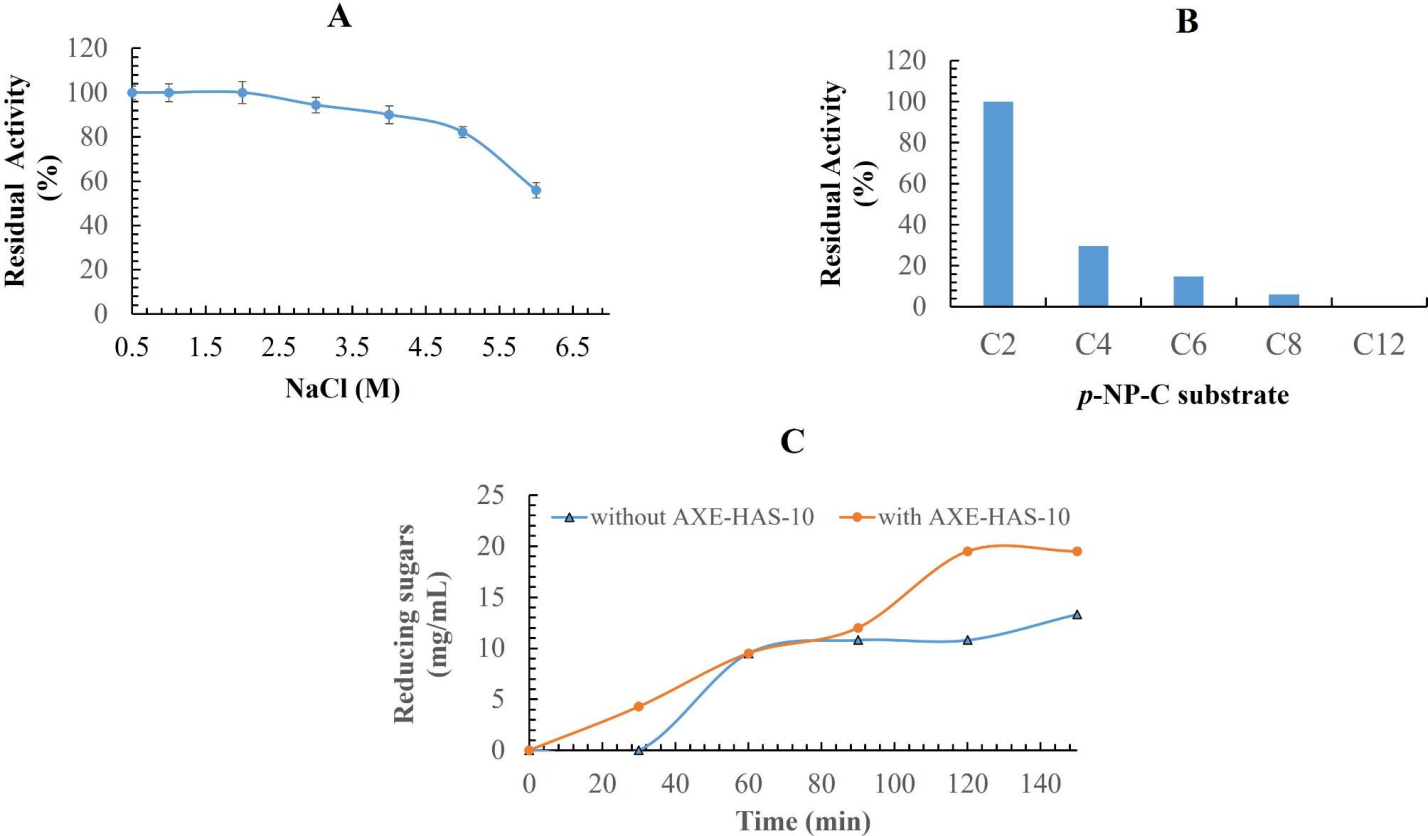
A: Effect of NaCl on the stability of purified to homogeneity AXE-HAS10 after pre-incubation of the purified AXE-HAS10 with effector for 30 min. Values are the mean of three readings ± standard error. B: Substrate specificity determination of AXE-HAS10 using *p*-nitrophenyl esters of different chain lengths: (*p*-nitrophenyl acetate (*p*-NP-C2,), *p*-nitrophenyl butyrate (*p*-NP-C4), *p*-nitrophenyl caproate (*p*-NP-C6), *p*-nitrophenyl caprylate (*p*-NP-C8), and *p*-nitrophenyl laurate (*p-*NP-C12)). Values are the mean of three readings ± standard error. C: Synergistic effect of AXE-HAS10 on beechwood xylan hydrolysis mediated by partially purified xylanase of *P. chrysogenum* Strain A3 DSM105774. Values are the mean of three readings ± standard error

The effect of various concentrations of NaCl on the stability of the purified AXE-HAS10 was studied as shown in Fig. 6 A. After pre-incubation of the enzyme for 30 min with varied concentrations of NaCl (1.0–4.0 M), there was no significant change in the values of retained enzyme activity, with the enzyme retaining around 100% of its original activity. In contrast, after 30 min of exposure to a higher concentration of NaCl (6.0 M), around 55.99 ± 1.9% of the initial enzyme activity could have been retained.

The PMSF did inhibit almost the majority of AXE-HAS10 activity at the two tested concentrations, 1.0 and 3.0 mM (Table S2).

The kinetic parameters for the hydrolysis of the most easily hydrolyzed substrate (*p*-NP-C2) by AXE-HAS10 are displayed in Table 1 and Fig. S1. AXE-HAS10 showed substrate affinity (*Km*), catalytic turnover (*kcat*), and catalytic efficiency (*k*_*cat*_*/K*_*m*_) of 0.096 mM, 3784 min^− 1^, and 39,426 min^− 1^.mM^− 1^, respectively.

The substrate specificity of AXE-HAS10 towar ds *p*-NP esters of lengths (C2-C12) was tested as shown in Fig. 6B. The highest activity of the enzyme was noticed on *p*-NP-C2. The retained activity of the enzyme on *p*-NP-C4, *p*-NP-C6, and *p*-NP-C8 was 29.68 ± 0.2, 14.87 ± 0.25, 6.03 ± 0.29%, respectively, relative to that of *p*-NP-C2 (set as 100%). No enzyme activity could be recorded using *p*-NP-C12 as a substrate.

The simultaneous usage of AXE-HAS10 and *P. chrysogenum* A3 DSM105774 β-xylanase exhibited 1.44 fold higher synergistic hydrolysis of beechwood xylan than the single utilization of β-xylanase (Fig. 6 C).

### Structure prediction of AXE-HAS10

**Fig. 7:**
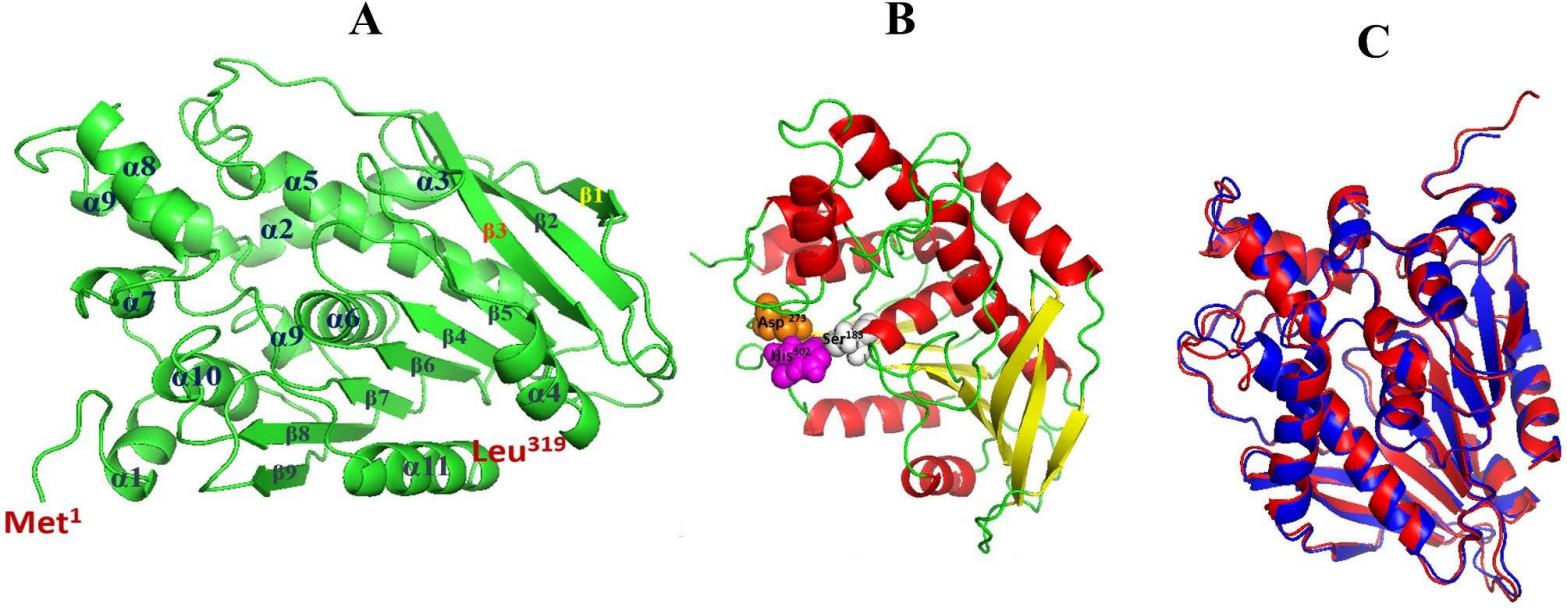
A: Predicted 3D structure of AXE-HAS10 built up with the MODELLER of LMOETS3 program in cartoon view showing the α /β fold structure (9 β-sheets and 11 α–helices), antiparallel β1 and β3 sheets to the rest β-sheets, Met^1^ (N-terminus), and Leu ^319^ (C-terminus). The view is for AXE-HAS10 in the monomeric state. B: Predicted 3D model in a cartoon view showing the catalytic triad at Ser ^183^, Asp ^273^, and His ^302^. C: Superimposed cartoon view of the 3D backbones of AXE-HAS10 ( blue) and 6fkx_A PDB template ( red) with TM- score and RMSD value of 0.988 and 0.83, respectively

The online Local Meta-Threading Server was used to predict the 3D structure of AXE-HAS10 (Fig. 7 A) (LOMETS version 3.0). The top three threading templates chosen by the LOMETS3 from 110 (= 11 × 10) templates were acetylxylan esterases from the desert metagenome (PDB: 6FKX), *Thermoanaerobacterium* sp. JW/SL YS485 (PDB: 3FCY), and *Paenibacillus* sp. R4 (PDB: 6AGQ). The highest normalized Z-score of 14.22 inferred a good alignment between the selected template (PDB: 6FKX_A) and the query sequence (AXE-HAS10). As a rule of thumb, a normalized Z-score of 1.0 indicates that the template and query are in good alignment. The catalytic triad (Ser^183^-Asp^273^-His^302^) of AXE-HAS10 was visualized in the 3D predicted model (Fig. 7B). The LOMETS3 MODELELR’s 3D predicted model of AXE-HAS10 was then submitted to the TM-align structural alignment programme in order to match the 3D projected model to all structures in the PDB collection. The top 10 proteins from the PDB collection with the closest structural similarity to the predicted model with the highest TM-score were discovered using the TM-align structural alignment software (Studer et al., 2020). Because of their structural similarities, these top 10 proteins usually have functions that are comparable to the target. When compared to other scores achieved with the remaining top 10 protein templates, the best selected template from the PDB collection was acetylxylan esterase of the desert metagenome (PDB: 6FKX_A), which had the greatest TM-score (0.988) and the lowest RMSD (root mean square deviation) value (0.83). The TM-score has a value of in most cases (0, 1). The TM-score of 1.0 indicates that the two 3D structures (i.e., the PDB structure template: 6FKX_A and the predicted model of AXE-HAS10) are identical. Furthermore, as shown in Fig. 7 C, the TM-align structural alignment tool created an ideal superposition of the two structures (i.e., the template with PDB: 6FKX_A and the predicted model of AXE-HAS10). The 3D predicted structure of AXE-HAS10 verified its typical α/β-fold hydrolase nature with 9 β-sheets and 11 α-helices. Additionally, β1 and β3 sheets were shown antiparallel to the rest β-sheets (Fig. 7 A).

## Discussion

More research is needed to determine the precise nature of microbial AXEs in terms of amino acid sequence, function, structure, biochemical characteristics, and substrate selectivity. In this context, the *AXE* gene from the locally obtained unique *Halalkalibacterium halodurans* strain NAH-Egypt was cloned, heterologously expressed, characterized, and *in silico* sequenced at the protein level. At NCBI, there are just two whole genome sequence (WGS) records for *A. halodurans* strains, C-125 and LB-1. Despite the putative gene of *AXE*, it was only found in the genome of *A. halodurans* C-125, not the LB-1 strain. Furthermore, there is only one report on *A. halodurans* C-125 recombinantly expressed AXE (BhAXE) in *E. coli* (Min-Jeong et al., 2020). Kim’s study did not cover either thorough biochemical characterization of recombinantly expressed BhAXE in *E. coli* nor *in silico* sequence analysis at the protein level (Min-Jeong et al., 2020). As a result, it was worthwhile to compare the properties of recombinantly expressed AXE (AXE-HAS10) from a locally obtained unique strain designated *A. halodurans* NAH-Egypt with those of recombinant BhAXE from *A. halodurans* C-125 previously produced in *E. coli* (Min-Jeong et al., 2020).

*In silico* sequence analysis of AXE-HAS10 revealed its affiliation to family CE7. So far, there are seven members of microbial AXEs in CE7 that are well characterized with determined crystal structures deposited in the Protein Databank (PDB) under the accession numbers: 3FVR_A, 1L7A, 7CUZ, 3FCY, 1LVQ, 6AGQ, and 6FKX from *B*. *pumilus*, *B*. *subtilis*, *Lactococcus lactis*, *Thermoanaerobacter saccharolyticum*, *Thermotoga maritima*, *Paenibacillus* sp., and a metagenome library, respectively. According to the classification of CAZy Server (Brandi et al. 2009), the previously reported signature motifs of CE7 (–RGQ–, –GxSxG– and HE) were localized at amino acid positions (118–120), (185–189), and (303–304), respectively, in the primary structure of AxeB (AHG97602.1), isolated from the termite hindgut metagenome (Mokoena et al. 2015). The signature- motifs of –RGQ–, –GXSQG–, and HE of CE7 were found in *B. pumilus* AXE (3FVR_A) at amino acids positions 118–120, 179–183, and 298–299, respectively (Degrassi et al. 2000).

It has been reported that the serine residue involved in the signature motif G-x-S-x-G is the catalytic serine. This serine residue helps attack the carbonyl carbon atom of the ester bond (Bornscheuer 2002; Mokoena et al. 2013). Based on the multiple sequences analysis, it was deduced that Ser^183^, Asp^273^, and His ^302^, in AXE-HAS10 did form the catalytic triad residues. Similarly, the catalytic triad residues in AXeA (AHG97601.1), AXeB (AHG97602.1), and AXE (AGF25253.1) from termite hindgut metagenome library (Mokoena et al. 2015), termite hindgut metagenome library (Mokoena et al. 2015)d *subtilis* CICC 20,034 (Tian et al. 2014) were localized as follows: Ser^182^-Asp^272^-His^301^, Ser^187^-Asp^273^-His^303^, and Ser^181^-Asp^269^-His^298^, respectively.

Likewise, AXE-HAS10 and *Al*AXEase from *Arcticibacterium luteifluviistationis* SM1504^T^ were severely inhibited by PMSF but at a much higher concentration (10 mM) of PMSF (Zhang et al. 2021). This would in turn suggest that AXE-HAS10 is a serine hydrolase.

With the online programme SignalP 5.0, no signal peptide was detected in the AXE-HAS10 primary amino acid sequence. It was reported that AXEs lacking signal peptides could be secreted due to internal signal peptide. Contrary to this speculation, AXEs lacking signal peptides could be intracellular enzymes rather than secretory enzymes with internal signal peptides (Degrassi et al. 2000; Walter et al., 1997). The recombinant AXE-HAS10’s experimentally measured molecular mass (41.39 kDa) was somewhat higher than the theoretical molecular mass (36.5 kDa). An additional sequence of 42 amino acids produced from the pET-28a (+) vector could account for the extra 4.6 kDa added to the molecular mass of the expressed recombinant AXE-HAS10. The 4.6 kDa sequence (42 amino acids produced from the pET-28a (+) vector) had 33 amino acids flanking the AXE-HAS10 fragment on the N-terminal side and 9 amino acids flanking the AXE-HAS10 fragment on the C-terminal side (Tham et al. 2020; Abady et al. 2022). The experimentally determined molecular mass of BhAXE from *B. halodurans* C-125, on the other hand, was completely consistent with that calculated theoretically (36 kDa). This could be due to the different expression vectors employed in the two studies: pHCXHD in Kim’s work (Min-Jeong et al., 2020) and pET-28a (+) in this study. The experimental molecular weight of AXE-HAS10 was in good agreement with the range of molecular weight of AXE(s) previously reported (27.0–41.0 kDa) (Table 2).

As a general principle, determining an enzyme’s physico-biochemical properties is a critical essential factor in the agenda of prospective industrialization for an enzyme’s ultimate and efficient exploitation.

In this study, AXE-HAS10 showed the greatest activity at pH 8.5 and a temperature of 40 ^o^C, respectively. Previously characterized AXEs showed a discrepancy in their optimal pH, temperature, and stability profiles as well. BhAXE exhibited its optimal activity at a pH and temperature of 8 and 50 ^o^C, respectively. After 15 h of incubation, AXE- HAS10 retained 100% of its activity at pHs 7.0–9.0. After 2 h of incubation, the retained activity of AXE-HAS10 at 35 and 40 ^o^C was ~ 80 and ~ 50%, respectively (Fig. 4D). For BhAXE, there was no obvious information in the study of Kim et al. about its thermal and pH profile stability (Min-Jeong et al., 2020).

Obviously, AXE-HAS10 is a cold-adapted AXE as it could display substantial activity (20, 30, 40, ~ 42, 50, 70, 100, 60, and ~ 0.0%) over a wide range of temperatures, 5, 10, 15, 20, 25, 30–35, 40, 45, and 55 ^o^C, respectively (Fig. 4 C). Moreover, the poor thermostability pattern of AXE-HAS10 (Fig. 4D) would greatly underpin its cold-adept nature. The cold-adapted nature of *(A) halodurans* NAH-Egypt was previously reported for estHIJ (Noby et al. 2020). The pH stability profile of AXE-HAS10 revealed its alkali-stability property. The difference between BhAXE and AXE-HAS10 could be attributed to the enzyme source strain difference and the ecological niche of the two producer strains: *(B) halodurans* C-125 (previously isolated from deep-see sediment) and *A. halodurans* NAH-Egypt (previously isolated from Natroun Valley, Egypt) (Noby 2020). Upon comparing the pH-temperature profile of other previously reported AXE(s) (Table 2) with that of AXE-HAS10, the pH and temperature optima spanned from 5.5 to 8.5 and 40–90 ^o^C, respectively.

**Table 2:** Comparison between some reported AXE(s) and AXE-HAS10 regarding biochemical properties

Microbial source	Enzyme designation	Expression host	a.a^a^	Optimal pH	Optimal Temp^b^. (^o^C)	M.W^c^ (kDa)	CE Fam.^d^	PDB accession number	Seq. Iden. (%) with AXE-HAS10	Activity at lowest temp (^o^C)	Thermost-ability	Kinetic Parameters on *p*-NP-C2	Reference
*Ochrovirga pacifca*	rAXE	*E. coli*	287	8.3	50	31.75	N. A	N. A	16.2	40% activity at 25 ^o^C	100% activity at 45 ^o^C for 2 h	N. A	Hettiarachchi et al. 2019
*Lactobacillus antri*	LaAXE	*E. coli*	323	7.0	50	37.00	CE7	N. A	37.8	65% activity at 30 ^o^C	N. A	N. A	Kim et al. 2020
*Bacillus halodurans* C-125	BhAXE	*E. coli*	319	8.0	50	36.00	CE7	N. A	100	45% activity at 30 ^o^C	N. A	N. A	Kim et al. 2020
*Thermoanerobacterium* sp.	rAXE1	*E. coli*	963	7.0	75–80	32.00	CE7	3FCY	63.3	N. A	60% activity at 75 ^o^C for 3 h	N. A	Shao and Wiegel 1995 & Lorenz and Wiegel 1997
*Clostridium saccharobutylicum*	EST1051	*E. coli*	307	7.0	40	35.00	CE7	N. A	40.0	30% activity at 4 ^o^C	60% activity at 60 ^o^C for 24 h	*Km* (0.923 mM)	Xu et al. 2021
Termite hindgut metagenome	AXEA	*E. coli*	325	7.5	40	40.00	CE7	N. A	54.9	30% activity at 30 ^o^C	50% activity at 60 ^o^C for < 1 h	*Km* (0.1 mM)	Mokoena et al. 2015
Termite hindgut metagenome	AXEB	*E. coli*	322	7.5	40	40.00	CE7	N. A	32.6	30% activity at 30 ^o^C	50% activity at 60 ^o^C for < 1 h	*Km* (0.23 mM)	Mokoena et al. 2015
*Halalkalibacterium halodurans* NAH-Egypt	AXE-HAS10	*E. coli*	319	8.5	40	41.39	CE7	N. A	100	22% activity at 5 ^o^C	100 & 82% activity at 35 ^o^C for 1 and 2 h, respectively	*Km* (0.096 mM)	This study
*Bacillus pumilus*	AXE	*E. coli*	320	8.0	55	40.0	CE7	2xlb, 3FYT, and 3FVT	38.0	N. A	50% activity at 60 ^o^C for 1 h	*Km* (1.54 mM)	Degrassi et al. 2000 Degrassi et al. 1998
*B. subtilis*	AXE	*E. coli*	318	8.0	50	35.6	CE7	1L7A, 1ODS, and 1ODT	36.8	65% activity at 30 ^o^C	100% activity at 40 ^o^C for 2 h	N. A	Tian et al. 2014
*Thermotoga maritima*	AxeA	*E. coli*	336	6.5	90	37.0	CE7	1VLQ, 3M81, 3M82, and 5GMA	31.6	20% activity at 30 ^o^C	50% activity at 98 ^o^C for 13 h & 90 ^o^C for 67 h	*Km* (0.12 mM)	Drzewiecki et al. 2010
*Penicillium chrysogenum* P33	PcAxe	*Pichia pastoris*	267	7.0	40	31.6	CE1	N.A	15.1	58% activity at 20 ^o^C	< 20% activity at 80 ^o^C for 1 h	*Km* (0.465 mM)	Yang et al. 2017
*Volvariella volvacea*	VvaxeII	*E. coli*	253	7.0	60	27.8	CE4	N.A	3.1	N.A	N. A	N. A	Liu and Ding 2009
*Aspergillus oryzae*	rAoAXE	*Pichia pastoris*	276	6.0	45	31.0	CE1	N.A	20.4	75% activity at 30 ^o^C	25% activity at 50 ^o^C after 1 h	N. A	Koseki et al. 2006
*Aspergillus niger*	AXE	*Aspergillus niger*	277	5.5-6.0	50	30.4	CE1	N. A	24.3	N. A	95% activity at 50 ^o^C for 2 h	N.A	Kormelink et al., 1993
*Streptomyces lividans* IFA43	AxeA	*Streptomyces lividans* IFA43	309	7.5	70	34.0	CE4	3COO	35.7	10% activity at 30 ^o^C	50% activity at 72 ^o^C for 15 min	N.A	Dupont et al. 1996

a: amino acids residues, b: temperature, c: experimental molecular weight, d: carbohydrate esterase family

N.A: not available

The ratio of flexible residues and their arrangement around the active site or across the protein structure could explain the differences in thermal stability (Marx et al. 2007). The ratio and distribution of Arg, Lys, Met, and Gly have been shown to play an important role in imposing cold-adeptness on cold-adapted enzymes (Marx et al. 2007; Fu et al. 2013). There are no reports on cold-adapted AXEs accessible, except for two that mention AlAXEase: AXE from *Arcticibacterium luteifluviistationis* SM1504T (Zhang et al. 2021) and PbAcE (PDB: 6AGQ) from *Paenibacillus* sp. R4 (Mavromatis et al., 2018). As a result of the scarcity of publications on cold-adapted AXEs, our cold-adapted AXE-HAS10 would be compared to AlAXEase, a new CE family member, in terms of the content of both enzymes from Arg, Lys, Met, and Gly. AXE-HAS10 has a Met and Gly content of 3.13 and 7.2%, respectively, whereas AlAXEase has a Met and Gly content of 2.7 and 7.2%, respectively. As shown, the Met and Gly ratios in AXE-HAS10 and AlAXEase are almost quite similar throughout the whole protein structure. Conversely, AXE-HAS10 showed a greater ratio of Arg/Arg + Lys (0.58) when compared to that of ***Al***AXEase (0.275). Despite this difference in Arg/Arg + Lys ratio between AXE-HAS10 and ***Al***AXEase being almost quite similar, full residual activity was traced for both enzymes at 5 ^o^C after 1 h. In comparison to that of AXE-HAS10, cold-adapted PbAcE (PDB: 6AGQ) from *Paenibacillus* sp. R4 had a slightly higher content of Met (3.1%) and Gly (8.39%) and a slightly higher content of Arg/Arg + Lys (0.607) (Park et al. 2018). However, the cold-adpation mechanism of proteins is case-by-case and should be explored for each protein by site-directed mutagenesis.

Reportedly, AXEs of microbial origin exhibit a special metal ion preference that would stimulate their activity. Unlike AXE-HAS10, AXE of *O. pacifca* was inhibited by 5 mM of Zn^2+^, Mn^2+^, and Ca^2+^ (Hettiarachchi et al., 2013). AXE from *B. subtilis* CICC 20,034 (Tian et al. 2014) was strongly inhibited by a very small concentration (1 mM) of Mn^2+^, Fe^3+^, Ca^2+^, and Zn^2+^. Like AXE-HAS10, an AXE from *P. chrysogenum* P33 (Hettiarachchi et al., 2017), was relatively stable in the presence of the following metal ions at high concentration (10 mM):Mn^2+^, Fe^3+^, Ca^2+^, and Zn^2+^. Despite the partial inhibitory effect (almost 60% retained activity) of some metal ions (i.e., Cu^2+^, Mg^2+^, and Mo^2+^) at 10 mM on AXE-HAS10, it was considered more metal ion stable than other AXEs from *O. pacifca* and *B. subtilis* CICC 20,034 that were inhibited by 1 and 5 mM of these metal ions. Although we do not yet have a strong explanation for the increased AXE-HAS10 activity in the presence of Mn^2+^ and Fe^3+^, prospective research of the Fe^3+^ and Mn^2+^: AXE-HAS10 ratio in this complex formation would be beneficial. It’s possible that the extraordinary increased activity in the presence of Fe^3+^ and Mn^2+^ is due to their binding to the amino acids that contribute to the active site. To test this hypothesis, a future study would need to co-crystallize AXE-HAS10 with these two ligands, Fe^3+^ and Mn^2+^. In conclusion, when compared to previously identified AXEs, AXE-HAS10 could tolerate a wide range of metal ions at relatively high concentrations (10 mM). This suggests that AXE-HAS10 is appropriate and efficient for usage in industrial settings with high metal ion loads.

AXE-HAS10 showed a promising profile regarding stability in the presence of a wide range of detergents (i.e., non-ionic, ionic, and cationic detergents). Incubation of AXE-HAS10 for 30 min with Triton x-100 at both concentrations of 0.01 and 0.02% exhibited a significant stimulatory effect with retained activity of 150 and 130%, respectively. The increased AXE-HAS10 activity in the presence of Triton-X100 in both concentrations could be owing to increased substrate availability to the active center associated with surfactant hydrophobic binding (Rao et al. 2013; Hua et al., 2013; Mitaku et al., 2002). Despite the inhibitory effect of both cationic and anionic detergents on AXE-HAS10, almost 85% of the activity was retained. Unfortunately, no data on the impact of detergents on the activity of CE7 AXEs could be described in the literature. The current data would strongly support the usage of AXE-HAS10 in industrial applications with a substantial detergent load. Regarding organic solvents, AXE-HAS10 demonstrated a relatively moderate stable profile towards hexane, glycerol, isopropanol, DMSO, methanol, and ethanol at 10% (v/v) after incubation for 30 min. Only one available report regarding the organic solvent stability of AXEs of CE7 was assigned to AXE from *B. subtilis* CICC 20,034 (Tian et al. 2014). In the study of Tian et al., the AXE showed robust stability (80–100%) towards 30% (v/v) of the organic solvents DMSO, methanol, acetone, isopropanol, benzene, toluene, and *n*-hexane after incubation for 24 h (Tian et al. 2014).

When compared to other previously reported AXEs, AXE-EST1051(Xu et al. 2021) and AXE of *O. pacifca* (Hettiarachchi et al. 2019), AXE-HAS10 appears to be more stable in the presence of high NaCl concentrations. AXE-EST1051 could retain 65% of its activity after pre-incubation for 20 min with 3 M NaCl (Xu et al. 2021). However, after 12 h of pre-incubation with 0.5 M NaCl, *O. pacifca* AXE could retain nearly 60% of its activity (Hettiarachchi et al. 2019). The current research clearly implies that AXE-HAS10 could be used in industrial environments with high salinity loading.

With regard to the effect of EDTA, the present findings reveal that AXE-HAS10 is a metallo-hydrolase similar to AXE from *Ochrovirga pacifica* (Hettiarachchi et al. 2019). Present data is in disagreement with the non-metallo acetyl xylan esterases *Al*AXEase from *Arcticibacterium luteifluviistationis* SM1504^T^ (Zhang et al. 2021) and FjoAcXE (Razeq et al. 2018).

AXE-HAS10 displayed very high activity toward *p*-NPA (C2) and had dramatically reduced activity towards *p*-nitrophenyl esters with carbon atoms from C2-C8 (Fig. 6B). Conversely, AxE of *B. subtilis* CICC 20,034 (Tian et al. 2014), AXE of *T. maritima* (Levisson et al. 2009), and AXE-EST1051 from a metagenomic library (Marx et al. 2007) demonstrated specific activity toward *p*-nitrophenyl esters with carbon atoms from C2-C4, C2-C12, and C2-C16, respectively. AxeA and AxeB activity from the termite hindgut metagenome (Mokoena et al. 2015) on *p*-nitrophenyl esters with carbon atoms from C2-C8 was found to be nearly identical to that of AXE-HAS10. A fold increase of 1.04, 1.25, and 2.39 in the *K*_m_ value of AxeA from *T. maritima* (Drzewiecki et al. 2010), AxeA and AxeB from a termite hindgut metagenome (Mokoena et al. 2015), on *p*-NPA-C2 when compared to that of AXE-HAS10. Hence, it would indicate the superior substrate specificity of AXE-HAS10 toward *p*-NPA (C2) relative to the aforementioned AXEs. In this perspective, AXE-HAS10 showed a superior *k*_*cat*_ (s^-1^) of 63.06 s^-1^ on *p*-NPA (C2) when compared to those of AxeA and AxeB from a termite hindgut metagenome (Mokoena et al. 2015), with 4.5 × 10^–11^ and 1.82 × 10^–11^ s^-1^, respectively. Conversely, the *kcat* of AXE-HAS10 was comparable to those of Axe from *T. maritima* (69.9 s^-1^) (Drzewiecki et al. 2010) and PbAcE from *Paenibacillus* sp. R4 (53.3 s^-1^) (Park et al. 2018).

The synergistic effects of some AXEs on the xylan polymer degradation were previously investigated with β-xylanase. Likewise, Kim and co-workers reported a fold enhancement of 1.44 upon simultaneous treatment of beech wood xylan with LaAXE (AXE from *L. antri*) and TnXNB. The AXE from *Volvariella volvacea* did succeed in enhancing the hydrolysis of xylan polymer by 1.4-fold when compared to the efficiency of xylan hydrolysis elicited by β-xylanases alone (Zheng et al. 2013). Similarly, the AXE from *O. pacifica* exhibited a 1.4-fold enhancement in the hydrolysis of beech wood xylan with a commercial β-xylanase (Hettiarachchi et al. 2019).

This is the first study to look at *Halalkalibacterium halodurans* strain NAH-cold-adapted Egypt’s AXE. Cold sensitivity, as well as detergent, metal ion, and halo-tolerance, would put AXE-HAS10 ahead of previously identified orthologous AXEs from other species. When combined with β-xylanase from *P. chyrysogenum* Strain A3 DSM105774, AXE-HAS10 produced synergistic xylan hydrolysis. Recombinant AXE-HAS10’s robust characteristics would support its potential in industrial applications. However, AXE-HAS10 should be subjected to a prospective study in order to highlight certain issues such as x-ray crystallography, site-directed evolution, and amino acid sequencing to unveil the structural-functional relationship encountered in this enzyme.

## Electronic supplementary material

Below is the link to the electronic supplementary material.

Supplementary Material 1: **Fig S1**: Lineweaver-Burk Plot performed by Hyper32 software for AXE-HAS10 upon using *p*-NP-C2 as the substrate

Supplementary Material 2: **Table S1**: PCR recipe and PCR conditions for amplification of full length ORF of xylan acetyl esterase from Alkalihalobacillus halodurans NAH-Egypt

Supplementary Material 3: **Table S2**: Effect of PMSF on AXE-HAS10 activity

## Data Availability

All data are available and included in the article.
