# Supplementary figures and images for: Recombinant acetylxylan esterase of Halalkalibacterium halodurans NAH-Egypt: molecular and biochemical study
Source: AMB Express. 2022 Oct 26;12:135. doi: 10.1186/s13568-022-01476-w (PMC9606172; doi:10.1186/s13568-022-01476-w)

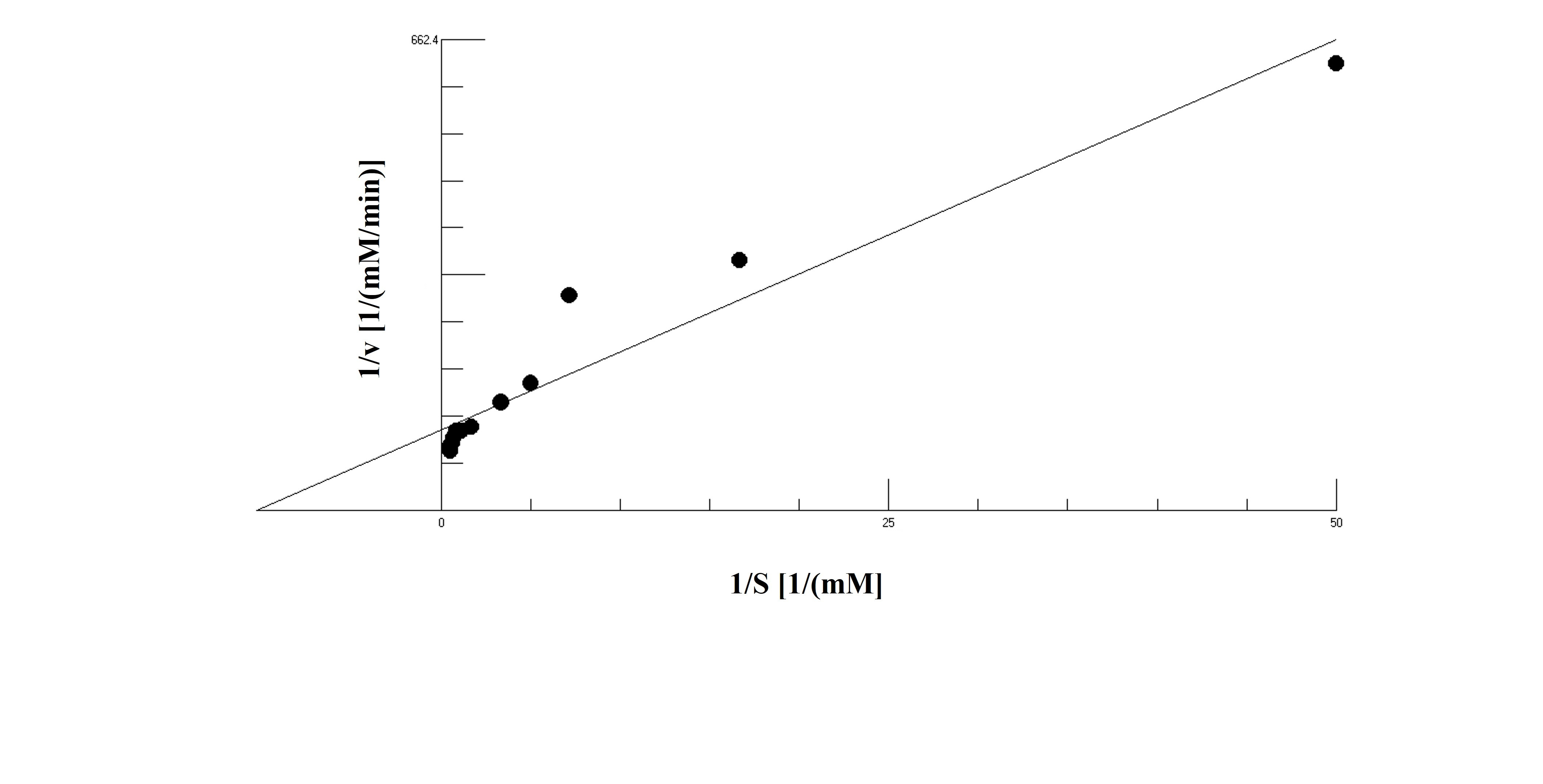

Supplement: Supplementary file 1 — Supplementary Material 1: Fig S1: Lineweaver-Burk Plot performed by Hyper32 software for AXE-HAS10 upon using p-NP-C2 as the substrate [file 13568_2022_1476_MOESM1_ESM.jpg]
