# Supplementary material for: Recombinant acetylxylan esterase of Halalkalibacterium halodurans NAH-Egypt: molecular and biochemical study
Source: AMB Express. 2022 Oct 26;12:135. doi: 10.1186/s13568-022-01476-w (PMC9606172; doi:10.1186/s13568-022-01476-w)
Supplement: Supplementary file 2 — Supplementary Material 2: Table S1: PCR recipe and PCR conditions for amplification of full length ORF of xylan acetyl esterase from Alkalihalobacillus halodurans NAH-Egypt [file 13568_2022_1476_MOESM2_ESM.docx]

**Table S1**: PCR recipe and PCR conditions for amplification of full length ORF of xylan acetyl esterase from *Alkalihalobacillus halodurans* NAH-Egypt

| **PCR recipe** | **PCR conditions** |
| --- | --- |
| Genomic DNA: 1 μL (30 ng)  AXE-HAS10-Fw-Prim: 2 μL (0.50 μM)  AXE-HAS10-Rv-Prim: 2 μL (0.50 μM)  My Taq^TM^ Master Mix (2X): 25 μL  Nuclease free water: 20 μL | 1 cycle: initial denaturation at 94 °C for 5 min;  30 cycles, each cycle:  denaturation at 94 °C for 30 s,  annealing at 58 °C for 30 s,  extension at 72 °C for 60 s,  1 cycle: final extension at 72 °C for 10 min. |
