# Supplementary material for: Recombinant acetylxylan esterase of Halalkalibacterium halodurans NAH-Egypt: molecular and biochemical study
Source: AMB Express. 2022 Oct 26;12:135. doi: 10.1186/s13568-022-01476-w (PMC9606172; doi:10.1186/s13568-022-01476-w)
Supplement: Supplementary file 3 — Supplementary Material 3: Table S2: Effect of PMSF on AXE-HAS10 activity [file 13568_2022_1476_MOESM3_ESM.docx]

Table S2: Effect of PMSF on AXE-HAS10 activity

| PMSF (mM) | Residual activity (%) |
| --- | --- |
| 1.0 | 8.7 ± 0.05 |
| 3.0 | 0.000 |
